# Academic Palliative Care Research in Portugal: Are We on the Right Track?

**DOI:** 10.3390/healthcare6030097

**Published:** 2018-08-12

**Authors:** Alexandra Pereira, Amélia Ferreira, José Martins

**Affiliations:** 1 Community Care Unit of Lousada, Rua de Santo Tirso 70, Meinedo, Lousada, 4620-848 Porto, Portugal; amelia.leite.ferreira@gmail.com; 2 Abel Salazar Biomedical Institute, R. Jorge de Viterbo Ferreira 228, 4050-313 Porto, Portugal; 3 Nursing School of Coimbra, 3046-841 Coimbra, Portugal; jmartins@esenfc.pt

**Keywords:** palliative care, end-of-life care, research

## Abstract

Background: The narrow link between practice, education, and research is essential to palliative care development. In Portugal, academic postgraduate publications are the main booster for palliative care research. Methods: This is a bibliometric study that aims to identify Portuguese palliative care postgraduate academic work published in electronic academic repositories between 2000 and 2015. Results: 488 publications were identified. The number of publications has increased, especially in the last five years. The most frequently used method was quantitative, healthcare professionals were the most studied participants, and psychological and psychiatric aspects of care comprised the most current theme. Practice-based priorities are financial costs and benefits of palliative care, awareness and understanding of palliative care, underserved populations, best practices, communication, and palliative care in nonhospital settings. Conclusion: The number of palliative care postgraduate academic publications has increased in Portugal in the past few years. There is academic production in the eight domains of quality palliative care and on the three levels of recommendation for practice-based research priorities. The major research gaps in Portugal are at the system and societal context levels.

## 1. Introduction

Changing demographic trends, such as an ageing population and increased life expectancy, in addition to medical and scientific advances accentuate the need to develop, evaluate, and research palliative care [1,2].

Palliative care is an approach for addressing the needs of individuals with life-threatening illnesses from a holistic, interdisciplinary perspective [3]. Estimates show that 19 million people need palliative care worldwide each year, 69% of which are older adults. Although the majority (78%) of adults in need of palliative care belong to low- and middle-income countries, the highest rates of those in need of palliative care per 100,000 adults are found in higher-income countries [4].

In recent years, palliative care research has evolved from care targeting patients with cancer to a care approach relevant for patients with diverse life-limiting conditions [5], therefore the scope of palliative care research is now wider. In spite of that, research in this field is still challenging, due to the sensitive nature of the topic and patients with complex and unstable symptoms [6]. Palliative care research has a fundamental role in informing evidence-based clinical practice, service development, education, and policy, and it is needed to improve service delivery and optimise patients’ quality of life [7,8].

With a population of 10 million, Portugal is the 7th most ageing country in the world [9] and is one of the countries with the highest rates of adults in need of palliative care [4]. Estimates show that Portugal needs 133 palliative care home teams, 102 hospital support teams, 28 palliative care units, and 46 hospices [10]. To date, according to the Portuguese palliative care directory, there are 20 palliative care home teams, 34 support teams, and 33 palliative care units. Comprising 18 districts and 2 autonomous regions, Portugal has an unequal distribution of palliative care teams: 10 districts don’t have home care teams and 3 districts don’t have a hospital support team or a palliative care unit [11]. Therefore, although Portugal is considered to have a generalized provision of palliative care [12], there still are inequities in the distribution of and access to palliative care [13].

Recently, the Portuguese palliative care strategic plan recognized education and research as two important vectors for palliative care development [14]. In 2003, the Council of Europe recommended that postgraduate training and education should be established in every country to ensure that every health professional is able to deliver palliative care in an insightful and culturally sensitive manner [15]. In Portugal, the first master’s course in palliative care emerged in 2002 at the University of Lisbon [16]. The offer of postgraduate education and training in this area has increased: five other master’s courses are now available [17]. The first doctoral program in palliative care was created in 2016 at the University of Porto.

The narrow link between practice education and research can enable the development of palliative care knowledge, identify research priorities, and contribute to evidence-based practice [18]. Education and practice are often identified as barriers to the development of palliative care [19,20,21], but, in fact, the gap between education research and practice might also be considered a problem that should be addressed as a complex and differentiated phenomenon [22], as significant knowledge gaps impede palliative care effectiveness. Educational research is essential to provide evidence for practice and practitioners can enrich research by posing adequate research questions. Thus, integrating the needs of palliative care practitioners with scientific expertise is likely to generate proposals for innovative studies that will ultimately improve practice [23]. In 2015, 10 recommendations for palliative care research to address knowledge gaps were published. These practice-based research priorities were clustered into three categories: (1) research to improve individual-level palliative care practice, (2) research to improve system-level palliative care practice and capacity, and (3) research on societal context for palliative care. These categories are mapped onto three domains that palliative care aims to affect: (1) the care that practitioners provide to patients and families, (2) palliative care organization and delivery, and (3) the popular, political, and social understanding and reception of palliative care [23].

Country reviews and bibliometric studies of palliative care research are motivated by a recognition of the importance of evidence in supporting decision-makers to meet the challenges that palliative care faces [24]. This is a current practice in research in different countries [24,25,26]. In Portugal, academic postgraduate publications are still the main booster for palliative care research, and thus, this bibliometric study aims to identify all Portuguese palliative care postgraduate academic works published in electronic academic repositories between 2000 and 2015 and to analyse their alignment with the internationally identified research priorities in palliative care. The research questions are:

(1) How many postgraduate academic works related to palliative care were published between 2000 and 2015 in Portugal? By which health professions?

(2) How many studies were undertaken?

(3) What designs were used and what populations were studied?

(4) What areas/themes were studied? Are they aligned with the identified research priorities in palliative care?

## 2. Materials and Methods

### 2.1. Design

This is a bibliometric study. Bibliometric methods are established as scientific specialties and are an integral part of research evaluation methodology, especially within the scientific and applied fields [27]. Bibliometrics is the application of quantitative analysis and statistics to publications [28], in this case, postgraduate academic research. We have chosen this method because we intend to obtain an overview of Portuguese postgraduate academic research related to palliative care. This way we can highlight a lack of evidence in certain areas that might help researchers to develop subsequent studies. Therefore, we haven’t made a critical appraisal of the articles or a synthesis of findings as conventionally required. The ethical procedures were guaranteed through rigorous methodology compliance and respect for the ethical principles that guide health research.

### 2.2. Search Strategies

This bibliometric study was undertaken to identify palliative care postgraduate academic research produced in Portugal. The following academic repositories were researched: Catholic University of Portugal, Coimbra Nursing School, Fernando Pessoa University, ISCTE-IUL, ISPA, Lusíada University, Lusófona University, Open University, Polytechnic Institute of Bragança, Polytechnic Institute of Castelo Branco, Polytechnic Institute of Oporto, Polytechnic Institute of Santarém, Polytechnic Institute of Viana do Castelo, Polytechnic Institute of Viseu, RCAAP, Technical University of Lisbon, New University of Lisbon, University of Algarve, University of Aveiro, University of Azores, University of Beira Interior, University of Coimbra, University of Évora, University of Lisbon, University of Oporto, and UTAD. The search terms used were “palliative care” or “end-of-life care” or “terminal care” in the following repository fields: subject or description or keyword.

### 2.3. Inclusion and Exclusion Criteria

Publications were selected based on the following inclusion criteria: (1) academic publications related to a master or a PhD, (2) academic publications relevant within the palliative care field, and (3) published between 2000 and 2015. Publications that were based on opinion or commentary or that were editorials, conference abstracts, or research papers were excluded.

### 2.4. Data Extraction

All postgraduate academic publications were exported to an Excel database and duplicates were removed. A data extraction protocol was developed. Results were analysed by two independent researchers to confirm the inclusion criteria. After the initial extraction by one of the researchers, the data extracted were cross-checked by the other researcher; consequently, all data were double-checked. When there was disagreement between the two researchers, a third researcher was invited to contribute an opinion to reach a consensus.

The data extracted from each included publication were as follows: author, author’s profession, author’s gender, year of publication, repository of publication, type of academic publication, research method, study participants, setting of care, and themes. Themes were decided a priori and were categorized after reading the abstracts according to the eight domains of quality palliative care: structure and processes of care; physical aspects of care; psychological and psychiatric aspects of care; social aspects of care; spiritual, religious, and existential aspects of care; cultural aspects of care; care of the imminently dying patient; ethical and legal aspects of care [29]. During the data extraction process, we added four other categories: lived experience of patients, lived experience of caregivers, lived experience of health professionals, and study of specific groups (for instance: diabetic patients, dementia patients, children). Recommendations for practice-based research priorities for palliative care were used to classify all studies [23]. After all the data were extracted, all postgraduate academic publications were categorized by the region corresponding to the repository. The author’s profession was confirmed by research on the electronic registration of Portuguese professional orders.

## 3. Results

**Figure 1:**
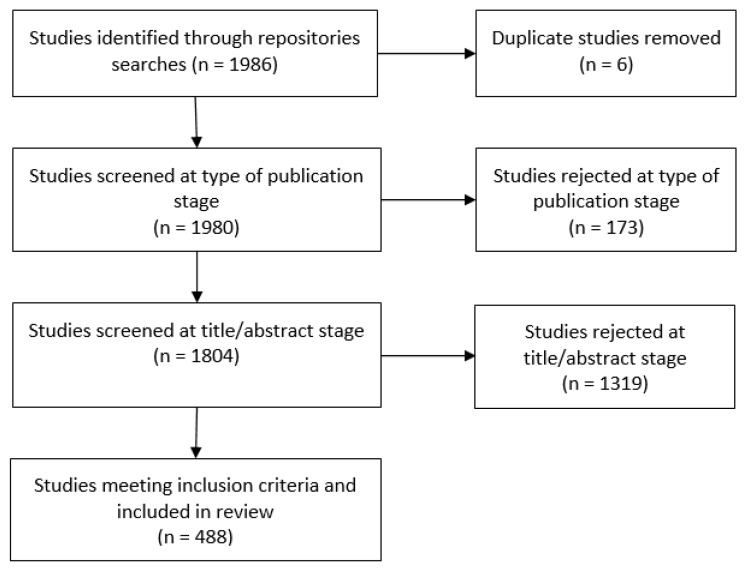
Selection and review process.

A flow diagram with the selection of cases is detailed in Figure 1. Overall, 28 repositories and 1980 studies were identified, after removing six studies that were duplicates. Also, 173 studies were excluded as they did not correspond to postgraduate academic publications. The titles and abstracts of the remaining 1807 studies were reviewed. This resulted in 1319 studies being excluded as they did not meet the inclusion criteria. The final review included 488 studies that were published in the electronic academic repositories during the period under examination.

From the 488 studies identified, 19.9% were published on the University of Oporto’s repository, followed by the Catholic University’s repository (17.8%), and the University of Lisbon’s repository (15.8%) (Table 1).

Regarding the region of the country where the university was located, there was a predominance of the northern (38.9%) and southern regions (37.7%) (Table 2).

The majority of authors were female (86.5%). Regarding the profession of the author, the majority were nurses (63.3%), followed by physicians (9.2%). It was not possible to identify the profession of the author in 8.6% of the studies (Table 3).

**Figure 2:**
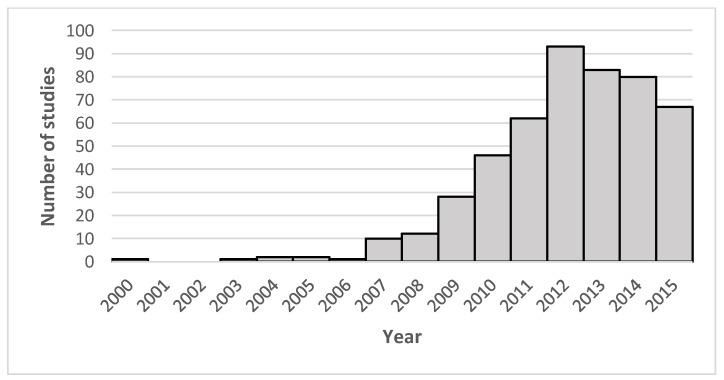
Number of studies per year (*n*).

There was an upward trend in the number of postgraduate academic studies published from 2009 to 2015 (94.1%). The oldest study was published in 2000. There was an absence of publications in 2001 and 2002 (Figure 2).

In terms of academic degree, 95.3% of the studies were published at the master’s degree level and 4.7% were published at the PhD degree level. In Portugal, a master’s degree can be obtained by a scientific dissertation, project work, or internship report [30]. In terms of type of academic publication, 68.9% were master’s dissertations, 18.6% were internship reports, 7.4% were research projects, and 5.1% were PhD theses (Table 4). Also, 51.0% of the postgraduate academic publications studies were not from a specific palliative care master’s or PhD degree, which shows that palliative care is a transversal theme of study in many healthcare-related courses.

Master’s dissertations and PhD theses were classified according to the research method described in the abstracts as shown in Table 5. Quantitative methods were used in 48.2% of the studies, followed by qualitative methods (31.0%).

In terms of study participants, the majority of publications involved healthcare professionals and/or healthcare students (32.1%), followed by patients (25.8%) and caregivers (17.7%). Also, a mixed population was studied in 7.5% of publications (Table 6). Only 3.3% of publications were related to paediatric palliative care.

Regarding the setting of research, we were able to classify 72.0% of publications. The investigation took place in a hospital setting in 47.9% of publications, followed by the community setting (11.1%), long term care setting (9.4%), hospital and community setting (2.2%), and resident home setting (1.4%).

The themes most frequently described in the 361 postgraduate academic publications were psychological and psychiatric aspects of care (19.4%), followed by structure and processes of care (19.1%) and physical aspects of care (15.5%). An additional nine themes were identified and we were unable to classify six publications (Table 7).

According to the level of practice-based research priority, most postgraduate academic publications were at the individual level (61.5%), meaning that the aim was to study the care that individual practitioners provide patients and families. The least represented level was the societal context for palliative care (5.0%). We were unable to classify 1.7% of the publications (Table 8).

The two most represented research priority recommendations were at the individual level of palliative care practice: symptom management (29.1%) and decision-making (23.3%). The third most represented was education and training for palliative care providers (13.0%) at system-level palliative care practice (Table 9).

## 4. Discussion

Country bibliometric studies of palliative care research are becoming a current practice in different countries as they support decision-making and can help researchers to identify potential lack of evidence in certain areas. We found three recent European studies, developed in Scotland [24], Ireland [25], and Sweden [26], that used a methodology similar to that in our study. Although it might seem odd to compare scientific publication production between countries, we have decided to do so in order to compare the Portuguese reality to countries that follow the same European guidelines yet have greater tradition and development in the palliative care field. There are also two Portuguese studies with which we will compare our results: one is from 2011 [31] and the other one, which is only nursing related, is from 2016 [32].

The findings from this bibliometric study show a considerable increase in palliative care postgraduate academic publications, particularly in the last five years. Today, there are almost nine times more postgraduate publications than the number reported until 2010 [31] and almost double the number of postgraduate publications reported in a bibliometric study until 2014, although this last one was only nursing related [32].

These results might be linked to several factors that have supported the development of palliative care in Portugal. Firstly, there has been an increase in the availability of postgraduate education related to palliative care and the possibility to join various scholarship programs. Secondly, there has been profuse public discussion surrounding palliative care in Portugal: TV debate programs have been made and research results have been diffused through various media channels. Thirdly, several government initiatives and laws have been implemented, including a recent publication of the Portuguese palliative care strategic plan that aims to foster awareness of the importance of palliative care and the creation of palliative care teams [14]. Lastly, there have been private funding initiatives to open palliative care home teams. These two last factors are also related to job market opportunities and might have had a direct influence on the increase in the number of people seeking specific palliative care education.

The fact that the majority of publications are from the north and south universities’ repositories is not surprising, as the two largest Portuguese cities are located in those regions and the majority of specific palliative care postgraduate courses are also offered there. Furthermore, there is evidence that postgraduate academic research is originating from different types of health courses, and, in similar vein, palliative care research papers are being published in a range of journals [33]. These results show that palliative care is an area of interest for a large portion of healthcare professionals from different backgrounds, even if the master’s or PhD course they are attending is not directly linked to the palliative care field.

The authors were predominantly nurses, followed by physicians. This is consistent with other Portuguese results [32]. The Swedish review showed the same tendency [26]. Although the composition of palliative care teams varies depending on needs and resources, the presence of physicians and nurses is constant and essential. The relation between nursing and palliative care is not new. In fact, Virginia Henderson said that palliative care “was the essence of nursing” [34]. Perhaps the fact that palliative care and nursing both maintain a commitment to the care of the whole person [35] may attract nurses to pursue the study of palliative care. Also, in Portugal, the recent government initiatives and the public discussion regarding palliative care has brought renewed attention to this area, which might be considered as a job opportunity. On the other hand, nursing postgraduate education has some tradition in Portugal: the master’s degree has existed since 1991 and the PhD degree since 2000. Even so, several other professions were found that reflect the multidisciplinary nature of palliative care. This multidisciplinary approach is fundamental to developing a consensus on the clinical definitions and guidelines for complex conditions and to provide comprehensive care [36]. This approach obliges their members to develop and share knowledge and skills that contribute to the overall functioning of the team [37].

In terms of design methods, the majority of the studies were descriptive and used a quantitative approach. Most quantitative studies were cross-sectional, using a small sample size. Also, few multicentre studies were found. This is consistent with other Portuguese results [31,32]. Similar results were obtained in Scotland and Ireland [24,25]. In Sweden, the qualitative approach was more commonly used [26]. Although the qualitative approach might be considered more holistic and humanized, in consideration of the nature of the studied themes it is understandable that the quantitative approach is more prevalent as palliative care research in Portugal is still a relatively new field. There is a lack of intervention studies in Portugal due to the fact that palliative care research can involve sensitive topics as well as ethical issues related to patients and families in vulnerable conditions [6]. In fact, these factors, in addition to the difficulty of achieving adequate sample sizes in a heterogeneous group of patients with chronic and incurable diseases, reduce the power of the studies and their follow-up periods, and so the “gold standard” of randomized trials is not necessarily applicable [8]. For these reasons, researchers usually employ alternative research methodologies, including observational studies with a large sample size and a valid methodology, in an attempt to improve palliative care research [38]. Commonly, postgraduate academic research is time constrained and usually limited in duration from a few months to one year in a master’s degree and up to three or four years in a PhD degree. Therefore, the utilization of a more accessible population can be a reason for why most publications have health professionals and students as study participants. Curiously, the Scottish review showed a preference for patient-related research [24]. There have also been few methodological studies (3.6%) conducted in Portugal. This type of study would be important for the development of adequate measurement instruments to improve the possibility to assess quality palliative-care-related indicators [39]. The lack of reliable instruments is one of the factors that hinder the assessment of quality indicators in palliative care [40].

Themes were categorized through abstract reading, and so the quality of abstracts may have influenced the categorization. It is important to reflect upon abstract quality in postgraduate academic research as it is an essential tool for the reader as it is for the author. The formatting and content of abstracts in academic research might be one barrier preventing a wider dissemination and use of research [41]. The three most prevalent themes that were found in our study were psychological and psychiatric aspects of care, structure of care, and physical aspects of care. From our point of view, and regarding the domains of quality palliative care [29], these themes are related to two dimensions: patient and family, and healthcare professionals. The first one is related to the following subthemes: patient and family needs assessment, symptom management, and grief/bereavement counselling. The second dimension is related to the following subthemes: education and training, and the emotional impact of work. Although similar country bibliometric studies use different classifications to categorize themes, it is possible to conclude that the results are similar. Symptom management was the most researched theme in Sweden [26], the second most researched in Ireland [25], and the third most researched in Scotland [24]. In the 2011 Portuguese study, medical care was the most researched theme [31]. We also discovered that only 5.5% of the publications were related to specific groups. In the Irish review this was the most researched theme [25]. In our study, 3.3% of the publications were related to children. In the Scottish review this was the 13th most researched theme [24]. Even so, we consider that these results are consistent with Portuguese practice, as paediatric palliative care is still an underdeveloped area in Portugal [42], and only in the past two years has attention been brought to this subject with the creation of a national group and the opening of the first paediatric palliative care unit in the north of Portugal.

The majority of publications were related to the hospital setting. Scotland has similar results, although the Portuguese results are more than double (47.9% versus 23.0%). In Sweden, the most common setting was home care. This might indicate that in Portugal, the healthcare system is hospital-centred. Recent studies show that, in spite of the fact that the majority of Portuguese people would prefer to die at home [43], most deaths occur in a hospital setting [44]. This subject has been discussed extensively in Portugal and the palliative care strategic plan highlights the importance of palliative care home teams, as a recent Cochrane review shows that home palliative care increases the chance of dying at home and reduces the symptom burden, without impacting on caregiver grief [45].

One of the aims of this study was to compare the results with the recommendations for practice-based research priorities for palliative care [23]. Although these recommendations were made in the United States, they have been adopted in the Portuguese palliative care strategic plan [14]. We found that there is postgraduate academic research production at the three levels of recommendations, but the majority is done at the individual level. This level includes the study of patients and families and the way that direct bedside practice is provided. In Portugal, palliative care emerged in the 1990s through pioneer initiatives and only a decade later did the first government initiative appear, so it was expected that most postgraduate academic research would be done at this level. Even so, a considerable number of studies were conducted at the system level of care practice and capacity, partly due to the research on education and training of palliative care providers. As we observed earlier, due to time constraints, many postgraduate academic research is done using health professionals and students as participants. Production at the societal context level is still low, whereby only 18 such studies were produced. Development of studies at this level of research is needed to improve the awareness and understanding of palliative care, to understand its financial cost and benefits, and to better understand underserved vulnerable populations. This is essential to improve the access to and distribution of palliative care in Portugal. Apart from the recommendations at the societal context level, major gaps in the postgraduate academic research in palliative care in Portugal are: best practices, communication, and palliative care in nonhospital settings.

## 5. Conclusions

This study provided baseline evidence of postgraduate academic research in Portugal over the last 15 years. The amount of postgraduate academic research has been increasing, especially in the last five years. The majority of postgraduate academic research was developed by nurses. A mix of research methods was identified, with a predominance of quantitative studies. Most postgraduate academic research is hospital-centred and the most studied population comprises healthcare professionals. In spite of that, several studies targeted patients. Most studied themes were the psychological and psychiatric aspects of care, structure and processes of care, and physical aspects of care. The current bibliometric study identified several palliative care research gaps, especially at the system and societal context levels. Practice-based priorities research in Portugal are: financial cost and benefits of palliative care, awareness and understanding of palliative care, underserved and vulnerable populations, best practices, communication, and palliative care in nonhospital settings.

The research undertaken was clearly limited by the fact that only electronic academic repositories were included. Although there might be a few publications in nonelectronic repositories, we believe that their inclusion would not be significantly relevant to the present study results. The keywords choice used on research might also be considered a limitation. Also, the poor quality of some abstracts might be considered a limitation during the data extraction.

We suggest that authors should improve the formatting and content of abstracts, as a poor-quality abstract can prevent a wider dissemination and use of research. We also suggest that domains of quality palliative care are used to classify themes/areas of focus in similar studies so that it is possible to obtain more comparable results in future research. A follow-up bibliometric study is recommended in a few years’ time.

## Figures and Tables

**Table 1:** Distribution of scientific production by repository (*n* and %).

Repository	*n*	%
Catholic University	87	17.8
Coimbra Nursing School	3	0.6
Fernando Pessoa University	4	0.8
ISCTE-IUL	6	1.2
ISPA	10	2.0
Lusíada University	1	0.2
University Lusófona	1	0.2
Open University	7	1.4
Polytechnic Institute of Bragança	16	3.3
Polytechnic Institute of Castelo Branco	49	10.0
Polytechnic Institute of Oporto	5	1.0
Polytechnic Institute of Santarem	8	1.6
Polytechnic Institute of Viana do Castelo	29	5.9
Polytechnic Institute of Viseu	12	2.5
RCAAP	10	2.0
Technic University of Lisbon	1	0.2
University Nova	7	1.4
University of Algarve	9	1.8
University of Aveiro	21	4.3
University of Azores	3	0.6
University of Beira Interior	7	1.4
University of Coimbra	18	3.7
University of Évora	1	0.2
University of Lisbon	1	0.2
University of Madeira	1	0.2
University of Oporto	97	19.9
UTAD	1	0.2
Total	488	100

**Table 2:** Distribution of scientific production by region (*n* and %).

Country Region	*n*	%
Azores	3	0.6
Centre	110	22.5
Madeira	1	0.2
North	190	38.9
South	184	37.7
Total	488	100

**Table 3:** Characteristics of authors.

Category	*n*	%
**Gender**		
Female	422	86.5
Male	66	13.5
Total	488	100
**Profession**		
Dentist	4	0.8
Gerontologist	6	1.2
Physician	45	9.2
Nurse	309	63.3
Occupational Therapist	5	1.0
Other	4	0.8
Pharmacist	3	0.6
Physiotherapist	12	2.5
Psychologist	35	7.2
Social worker	16	3.3
Sociologist	3	0.6
Speech Therapist	4	0.8
Unknown	42	8.6
Total	488	100

**Table 4:** Distribution of scientific production by type of academic publication (*n* and %).

Type of Academic Publication	*n*	%
Internship reports	91	18.6
Master’s dissertation	336	68.9
PhD theses	25	5.1
Research projects	36	7.4
Total	488	100

**Table 5:** Distribution of scientific production by type of research methods (*n* and %).

Type of Research Method	*n*	%
Measurement/methodology	13	3.6
Mixed methods	19	5.3
Other methods	10	2.8
Qualitative	112	31.0
Quantitative	174	48.2
Reviews	28	7.7
Unclassifiable	5	1.4
Total	361	100

**Table 6:** Distribution of scientific production by type of study participants (*n* and %).

Type of Study Participants	*n*	%
Caregivers	64	17.7
Caregivers and health professionals	6	1.7
Documentation	40	11.1
General population	1	0.3
Health professionals and/or students	116	32.1
Other	3	0.8
Patients	93	25.8
Patients and caregivers	6	1.7
Patients and health professionals	6	1.7
Patients, caregivers and health professionals	9	2.5
Unclassified	17	4.6
Total	361	100

**Table 7:** Distribution of scientific production by areas of focus (*n* and %).

Areas of Focus	*n*	%
Care of the imminently dying patient	9	2.5
Cultural aspects of care	2	0.6
Ethical and legal aspects of care	26	7.2
Lived experience of caregiver	26	7.2
Lived experience of health professional	39	10.8
Lived experience of patient	10	2.8
Physical aspects of care	56	15.5
Psychological and psychiatric aspects of care	70	19.4
Social aspects of care	13	3.6
Specific groups	20	5.5
Spiritual, religious, and existential aspects of care	15	4.2
Structure and processes of care	69	19.1
Unclassified	6	1.6
Total	361	100

**Table 8:** Distribution of scientific production by level of research priority recommendation (*n* and %).

Level of Research Priority Recommendation	*n*	%
Individual-level palliative care practice	222	61.5
System-level palliative care practice and capacity	115	31.9
Societal context for palliative care	18	5.0
Unclassified	6	1.7
Total	361	100

**Table 9:** Distribution of scientific production by research priority recommendation (*n* and %).

Research Priority Recommendation	*n*	%
**Individual-level palliative care practice**		
Communication	21	5.8
Decision-making	84	23.3
Symptom management	105	29.1
Best practices	12	3.3
**System-level palliative care practice and capacity**		
Nonhospital settings	28	7.8
Education and training for palliative care providers	47	13.0
Palliative care across the span of serious illness and the end of life	40	11.1
**Societal context for palliative care**		
Awareness and understanding of palliative care	11	3.0
Financial costs and benefits of palliative care	3	0.8
Underserved and vulnerable populations	4	1.1
Unclassified	6	1.7
Total	361	100
